# Immunosurveillance shapes the emergence of neo-epitope landscapes of sarcomas, revealing prime targets for immunotherapy

**DOI:** 10.1172/jci.insight.170324

**Published:** 2023-07-10

**Authors:** David O. Osei-Hwedieh, Abigail L. Sedlacek, Luis Mena Hernandez, Archibald Agyekum Yamoah, Swati G. Iyer, Kurt R. Weiss, Robert J. Binder

**Affiliations:** 1 Department of Immunology and; 2 Department of Medicine, University of Pittsburgh, Pittsburgh, Pennsylvania, USA.; 3 Department of Orthopaedic Surgery, UPMC Hillman Cancer Center, Pittsburgh, Pennsylvania, USA.

**Keywords:** Immunology, Adaptive immunity, Cancer, Immunotherapy

## Abstract

T cells recognize tumor-derived mutated peptides presented on MHC by tumors. The recognition of these neo-epitopes leads to rejection of tumors, an event that is critical for successful cancer immunosurveillance. Determination of tumor-rejecting neo-epitopes in human tumors has proved difficult, though recently developed systems approaches are becoming increasingly useful at evaluating their immunogenicity. We have used the differential aggretope index to determine the neo-epitope burden of sarcomas and observed a conspicuously titrated antigenic landscape, ranging from the highly antigenic osteosarcomas to the low antigenic leiomyosarcomas and liposarcomas. We showed that the antigenic landscape of the tumors inversely reflected the historical T cell responses in the tumor-bearing patients. We predicted that highly antigenic tumors with poor antitumor T cell responses, such as osteosarcomas, would be responsive to T cell–based immunotherapy regimens and demonstrated this in a murine osteosarcoma model. Our study presents a potentially novel pipeline for determining antigenicity of human tumors, provides an accurate predictor of potential neo-epitopes, and will be an important indicator of which cancers to target with T cell–enhancing immunotherapy.

## Introduction

Immunosurveillance mechanisms in tissues eliminate precancerous cells prior to formation of malignancies. This is supported by evidence from immunodeficient individuals where a much higher incidence of a variety of cancers has been reported compared with immunocompetent counterparts (1, 2). Empirical evidence from mice shows that effector mechanisms responsible for eliminating these emerging, nascent tumors primarily involve T cells and NK cells (3–6). However, these nascent tumors can develop mechanisms to escape surveillance, leading to pathology and clinical manifestations (7). As tumors progress, immunological pressure forces cancer cells to express fewer immunogenic epitopes that can be targeted, when compared with similar tumors emerging in the absence of such immunological pressure responses (4, 8).

Antigenic targets for T cells in the setting of mouse and human cancers are primarily mutated peptides, derived from processed parent proteins encoded by the mutated tumor genome, and presented by MHC (5, 8–14). The absence of these mutated antigens renders cancer immunosurveillance ineffectual and emerging tumors are not rejected (6). These mutated neo-epitopes are generally the determinants of tumor antigenicity and have been exploited clinically (15–17). However, the much-used in silico neo-epitope characterization, by half maximal inhibitory concentration threshold (IC_50_) of mutated peptide association to MHC, is problematic. These are the classically defined neo-epitopes (CDNs) (18), and their correlation with immunogenicity is extraordinarily poor; i.e., many CDNs are not immunogenic, and many neo-epitopes excluded as CDNs mount potent T cell immune responses (19–21). Consequently, when CDN is selected for cancer immunotherapy, the objective responses and clinical outcomes are often disappointing (20). In addition, mutational load by itself is not a predictor of tumor antigenicity (22). The differential aggretope index (DAI), which determines the differences in improved affinity of mutated peptides to bind MHC relative to their nonmutated peptides, has emerged as an excellent indicator of neo-epitope immunogenicity (8, 14, 18, 23). The DAI is based on prior observations, in multiple antigenic systems, demonstrating that immunogenicity of peptide antigens is enhanced when the stability of the peptide/MHC is increased (24, 25). In particular, when mutations in the anchor residues increase affinity of the peptide for MHC, the stability of the peptide/MHC is enhanced, resulting in improved TCR recognition (26). The alternative is also true; mutations that do not improve MHC binding do not improve TCR recognition (27). When DAI was first applied to MethA murine tumors, the measurements successfully identified specific MethA tumor rejection neo-antigens (14). Similar application of DAI to human cancers has identified neo-antigens in melanoma and advanced lung carcinomas (8, 23). Structural and crystallographic studies have subsequently uncovered a mechanism by which higher affinity results in increased TCR recognition (21).

We have previously shown that tumors emerging in immunodeficient mice had many immunogenic neo-epitopes as determined by DAI ≥ 8 (8). In contrast, tumors emerging in immunocompetent mice had almost exclusively mutated neo-epitopes with DAI < 8, representing classic tumor immunosurveillance. In addition to providing an important metric for selecting mutant neo-epitopes for immunotherapy of cancer, the DAI threshold is a vital indicator of tumor antigenicity because that provides targets for cancer immunosurveillance.

In this study, we analyzed freshly excised bone and soft tissue sarcomas from 122 patients for their antigenicity and immunogenicity by, respectively, measuring the DAI of all mutated neo-epitopes expressed on tumors and analyzing tumor-infiltrating T cells (TILs). We found that in patients with strong T cell responses in situ, including memory responses, as determined by TIL clonal expansion, emergent tumors had cleared neo-epitopes with high DAI. The converse was true; patients with poorer T cell responses had tumors expressing neo-epitopes with high DAI. Importantly, DAI threshold for immunosurveillance determined here with human tumors was strikingly similar to that previously found in mouse systems (8). Osteosarcomas, in particular, had many more neo-epitopes with high DAI compared with other sarcomas analyzed, and patients bearing these osteosarcomas had generally poorer TIL. The heat shock protein (HSP) receptor, CD91, has previously been shown to be a key molecule necessary for effective immunosurveillance of emerging tumors (8). CD91 dysfunction (by lack of expression or its mutation) leads to abrogation of tumor immunosurveillance, and CD91 expression strongly correlates with clinical prognosis in patients with cancer (8, 28). In reductionist experiments, CD91 has been shown to be necessary for cross-presentation of HSP-chaperoned peptides (29–32) and signaling linked to costimulation for adaptive T cell responses (33, 34). We show here that clonal expansion of TILs correlated with patients’ CD91 expression level, with only marginal differences in infiltrates of antigen-presenting cells. Our observations, particularly the expression of high-DAI neo-epitopes, suggested that osteosarcomas would be particularly amenable to T cell–based immunotherapy. We tested this using tumor-derived HSP-based immunotherapy, which primes robust tumor-rejecting T cell responses (35–37). We established a mouse model of metastatic osteosarcoma and observed that tumor-derived HSPs primed favorable antitumor T cell responses capable of rejecting a tumor challenge. In addition, treatment of mice bearing lung metastasis with tumor-derived HSPs as a monotherapy or in combination with checkpoint blockade profoundly prolonged survival. Our studies demonstrate a potentially novel pipeline for analyzing antigenic landscapes of tumors with the ability to distinguish the titrated nuances, a similarity of immunosurveillance mechanisms in murine and human systems, and provide potentially novel immunotherapeutic approaches for patients with cancer.

## Results

### Stratification of patient tumor samples.

**Figure 1:**
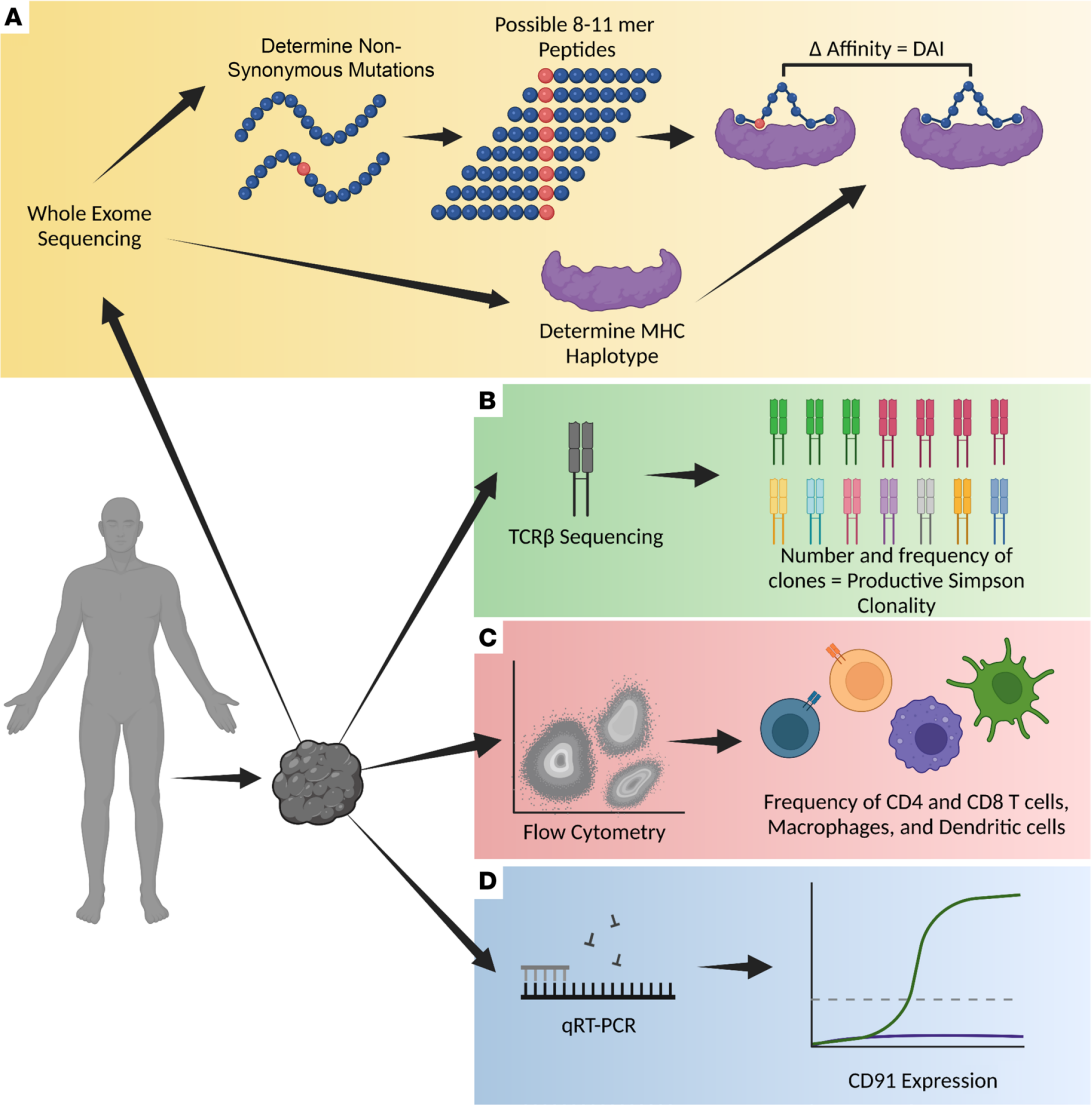
Analytical workflow pipeline to measure antigenicity and immunogenicity of sarcomas. Bone and soft tissue sarcomas from 122 patients were isolated and analyzed by the following mechanisms. (**A**) Whole-exome sequencing to determine DAI and each patient’s MHC haplotype for HLA-A, -B, and -C. (**B**) TCRβ sequencing to determine Productive Simpson Clonality (PSC) as a measure of T cell diversity. (**C**) Flow cytometry to determine the frequency of total immune cells, CD4^+^ T cells, CD8^+^ T cells, macrophages, and dendritic cells within the excised tumor sample. (**D**) Quantitative real-time PCR (qRT-PCR) to determine the mRNA expression level of CD91.

To investigate the antigenicity and immunogenicity of human sarcomas, we established an effective analytical pipeline (Figure 1). Freshly excised bone-proximal tumors from 122 patients (107 sarcomas and 15 carcinoma bone metastases) were taken through the pipeline to obtain information on mutational burden, MHC haplotype and neo-epitopes (Figure 1A), clonal expansion of TILs (Figure 1B), immune cell infiltrates of tumors (Figure 1C), and CD91 expression (Figure 1D). Sarcoma samples were part of the Musculoskeletal Oncology Tumor Registry and Tissue Bank (MOTOR) registry at the UPMC Hillman Cancer Center as described in Methods. All sarcomas are of mesenchymal origin but are generally classified into increasingly smaller pathological subtypes, of which there are over 50 (Supplemental Table 1; supplemental material available online with this article; https://doi.org/10.1172/jci.insight.170324DS1) (38). We placed our samples into 1 of 10 broad categories based on tissue origin of the primary lesion (Tables 1–3) mainly to obtain sufficient numbers of each tumor type for power calculations. Samples from patients with lesions of the bone, cartilage, or smooth muscle (Table 1) were analyzed by all parameters. Samples from patients with lesions of the skeletal muscle, adipose, and metastatic carcinomas (Table 2) were analyzed by whole-exome sequencing, flow cytometry, and qRT-PCR. Remaining samples of patients with sarcoma (Table 3) were analyzed by flow and qRT-PCR. Among the 107 patients with sarcoma, 3 were ultimately diagnosed with benign lesions and 3 with lesions suspected of being benign. These patients’ samples were analyzed exclusively by flow cytometry and were not included in the more in-depth analysis.

### Antigenic landscape variation of bone and soft tissue sarcoma.

**Figure 2:**
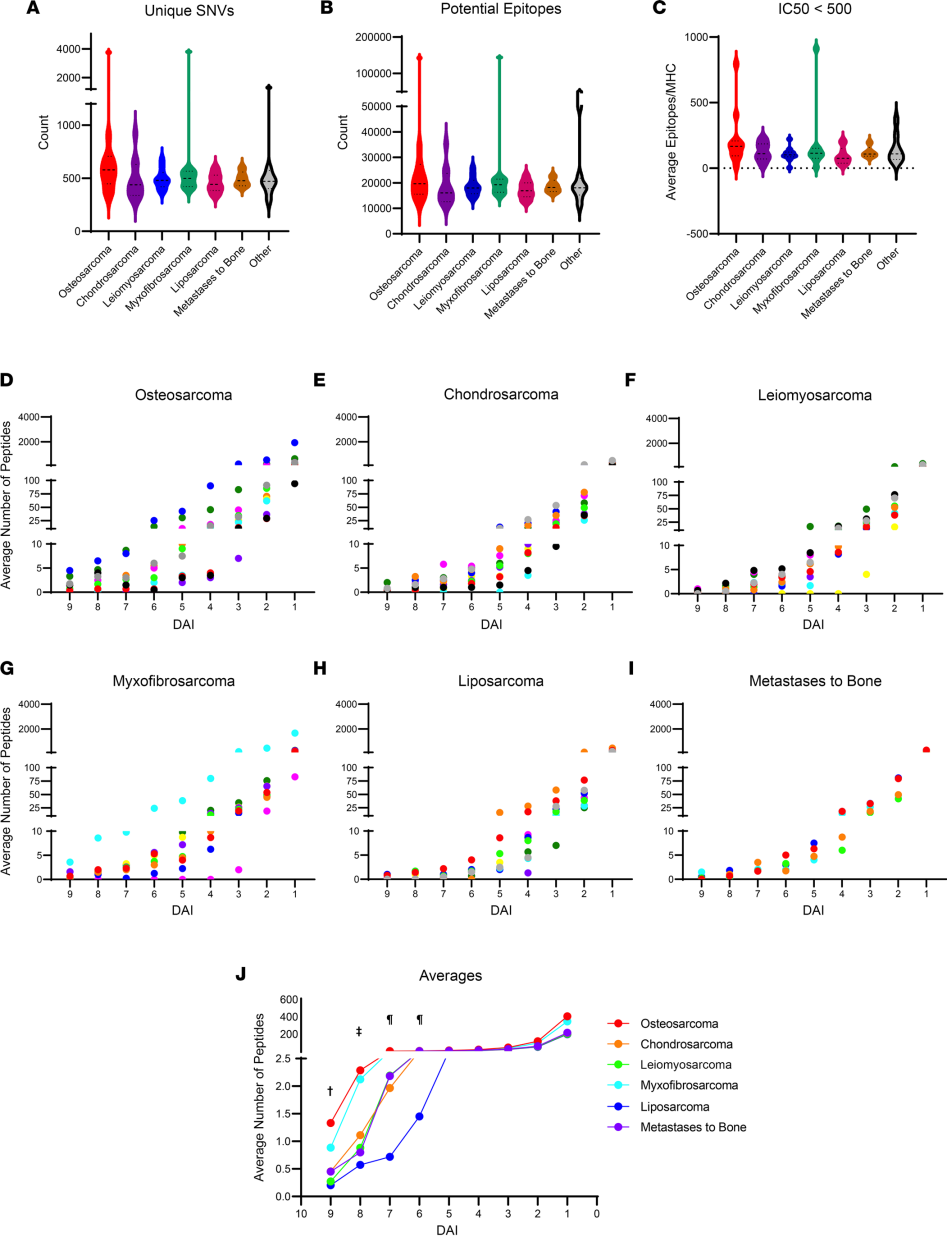
Genomic and mutational analysis and neo-epitope expression reveal differences in antigenicity of sarcoma subtypes. All samples analyzed by whole-exome sequencing were assessed for (**A**) total unique SNVs, (**B**) total potential epitopes derived from all SNVs, and (**C**) the average number of SNVs per MHC with an IC_50_ below 500. Data are presented as a violin plot with median and IQR as dashed lines. Whole-exome sequences were used to calculate DAI for all predicted epitopes as described in Methods (**D**–**I**). The average number of epitopes per HLA was plotted by grouped DAI values: 9 (>9), 8 (8.99–8), 7 (7.99–7),… 1 (1.99–1). Tumors were clustered by tissue of origin: (**D**) osteosarcoma (*n* = 11), (**E**) chondrosarcoma (*n* = 11), (**F**) leiomyosarcoma (*n* = 11), (**G**) myxofibrosarcoma (*n* = 9), (**H**) liposarcoma (*n* = 10), and (**I**) distal tumors that metastasized to bone (*n* = 5) (**J**). Average number of epitopes per HLA was plotted for each tumor type. Significance was determined by ANOVA (^†^ = osteosarcoma is significantly different from leiomyosarcoma**, liposarcoma**, and chondrosarcoma*; ^‡^ = osteosarcoma is significantly different from leiomyosarcoma* and liposarcoma*; ^¶^ = osteosarcoma is significantly different than liposarcoma*). *P < 0.05, **P < 0.01.

Whole-exome sequencing revealed that all 6 sarcoma types analyzed, on average, had comparable number of single nucleotide variations (SNVs) (Figure 2A) and number of unique epitopes predicted to be generated from each SNV (Figure 2B). We determined each patient’s MHC based on whole-exome sequencing data and predicted 8-, 9-, 10-, and 11-mer mutant peptides for each SNV using NetMHC33 (Figure 1A). There were comparable CDNs based on IC_50_ at <500 (Figure 2C). This result is consistent with prior observations by us and others that CDNs do not adequately capture antigenic variations of tumors (8, 18, 20, 21, 23). We next determined the DAI for neo-epitopes (Figure 1A) (8, 14, 18, 23). From the same 8-, 9-, 10-, and 11-mer mutant peptides for each SNV predicted to bind every MHC allele identified per patient, we calculated the DAI as described in Methods and obtained the number of neo-epitopes with DAI 1–1.99, 2–2.99, 3–3.99 …, and >9. Important differences emerged between tumor types. Osteosarcomas (Figure 2D) generally had a greater number of neo-epitopes with high DAI compared with all other tumor types tested (Figure 2, E–I), indicating that these tumors were more antigenic. Leiomyosarcomas and liposarcomas were particularly devoid of neo-epitopes with high DAI (Figure 2, F and H). Effector mechanisms of immunosurveillance that lead to rejection of tumors in mice target neo-epitopes with DAI ≥ 8 (8). When we considered this threshold for distinguishing between strong and weak neo-epitopes in human tumors, osteosarcomas presented significantly more neo-epitopes with DAI ≥ 8 than leiomyosarcomas or liposarcomas (as representative tumors with low antigenicity) (Figure 2J), suggesting that osteosarcomas are generally highly antigenic. We also observed that many of the high-DAI neo-epitopes on all tumor types were constituted preferentially with HLA-A and HLA-B over HLA-C (Supplemental Figure 1). This is consistent with previous observations that suggest while HLA-A/B are associated with neo-epitope presentation to, and activation of, CD8^+^ T cells, HLA-C is more strongly associated with interactions with killer Ig-like receptors expressed by NK cells (39), implicating these 2 immune effector cells in cancer immunosurveillance to varying degrees.

### Immunosurveillance of sarcomas is dependent on antigenicity.

**Figure 3:**
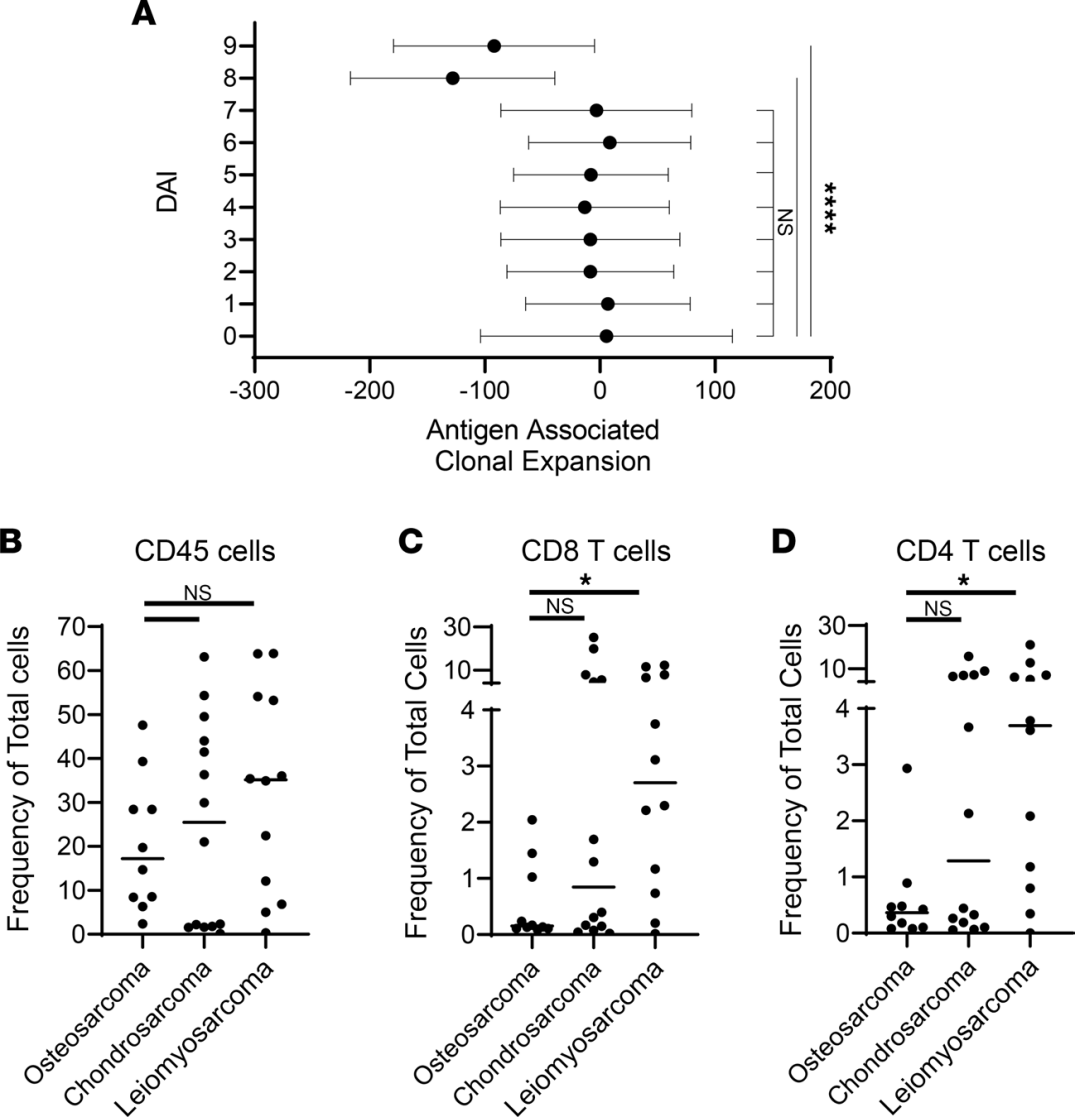
T cell responses in the tumor microenvironment alter the antigenic landscape of emergent tumors. (**A**) A subset of sarcomas (*n* = 39) were analyzed for their PSC score and DAI. DAI values were grouped 9 (>9), 8 (8.99–8), 7 (7.99–7),… 1 (1.99–1) and plotted against the number of antigens/tumor for each group. The resulting slopes from the regression analysis (Supplemental Figure 2) are plotted in a forest plot as antigen-associated clonal expansion. The frequency of (**B**) CD45^+^ cells, (**C**) CD8^+^ T cells, and (**D**) CD4^+^ T cells among total number of cells in the excised tumor was determined by flow cytometry and plotted for each indicated tumor type. (*n* = 10–14.) Significance was determined by 1-way ANOVA. *P < 0.05, ****P < 0.0001.

Based on DAI of neo-epitopes from Figure 2, we selected 3 sarcoma types (*n* = 39) of high (osteosarcomas), medium (chondrosarcomas), and low (leiomyosarcoma) antigenicity for further analysis. We examined T cell responses in patients with these 3 sarcoma types by sequencing the TCRβ of TILs (Figure 1B). The PSC score, a measure of TCR diversity of TILs, is a historical reflection of responses to an evolving neo-epitope landscape in an emerging tumor. PSC score ranges 0–1, where 0 is a completely diverse population of TCRs with no duplication and a score of 1 means the T cell population is monoclonal and has responded and expanded to a single antigenic peptide/MHC complex. The PSC score was then compared with the number of neo-epitopes at each DAI (1–1.99, 2–2.99, 3–3.99, 4–4.99...,and >9) in a patient’s tumor to obtain the antigen-associated clonal expansion as described in Methods. A highly negative correlation was observed when PSC was compared with number of neo-epitopes of high (≥8) DAI (Figure 3A). As expected, the correlation of PSC with the number of neo-epitopes of low (1–<8) DAI did not differ significantly from the correlation seen between PSC and epitopes with no change in affinity (DAI –0.99–0.99) (Figure 3A). This result indicates that patients with poor T cell responses (low PSC) had tumors that expressed significantly more high-DAI (≥8 or 9) neo-epitopes (Supplemental Figure 2). Conversely, patients with strong T cell responses (higher PSC) in the tumor microenvironment ended up with tumors expressing almost exclusively low-DAI (<8) epitopes (Figure 3A), negating the relationship (Supplemental Figure 2). The threshold for executing T cell responses in human cancers appears to be neo-epitopes with DAI ≥ 8, strikingly similar to results from the murine system (8). We immunophenotyped tumors by flow cytometry (Figure 1C) as described in Methods to examine frequency of T cells. There was overall no observable difference between infiltrating immune (CD45^+^) cells of the 3 different tumor types (Figure 3B). However, osteosarcomas were less infiltrated by CD8^+^ T cells compared with chondrosarcomas and leiomyosarcomas (Figure 3C), with the latter comparison being significant. This was also true for CD4^+^ T cells (Figure 3D). We were attentive to the fact that not all patients received chemotherapy and minimized its impact by obtaining tumor samples at least 1 month following the last treatment (Supplemental Table 1). Our analysis showed that there was no significant difference in immune cell infiltration between patients who received chemotherapy prior to tumor resection and those who had not (Supplemental Figure 3). The PSC score and T cell frequency data are consistent with one another and support the premise that patients with osteosarcomas mount poor antitumor immune responses, which allows for the emergence of tumor cells expressing strong antigens/neo-epitopes. Conversely, patients with leiomyosarcomas mounted strong T cell responses that led to emergence of tumor cells expressing few/no strong antigens/neo-epitopes. Chondrosarcomas presented a midrange of antigenicity (Figure 2B), sitting between osteosarcomas and leiomyosarcomas, and those patients also had an intermediate (biphasic) T cell pattern (Figure 3, C and D). This suggests our pipeline can distinguish a nuanced titration in antigenicity and cancer immunosurveillance.

### CD91 expression correlates with T cell immunity to sarcomas.

**Figure 4:**
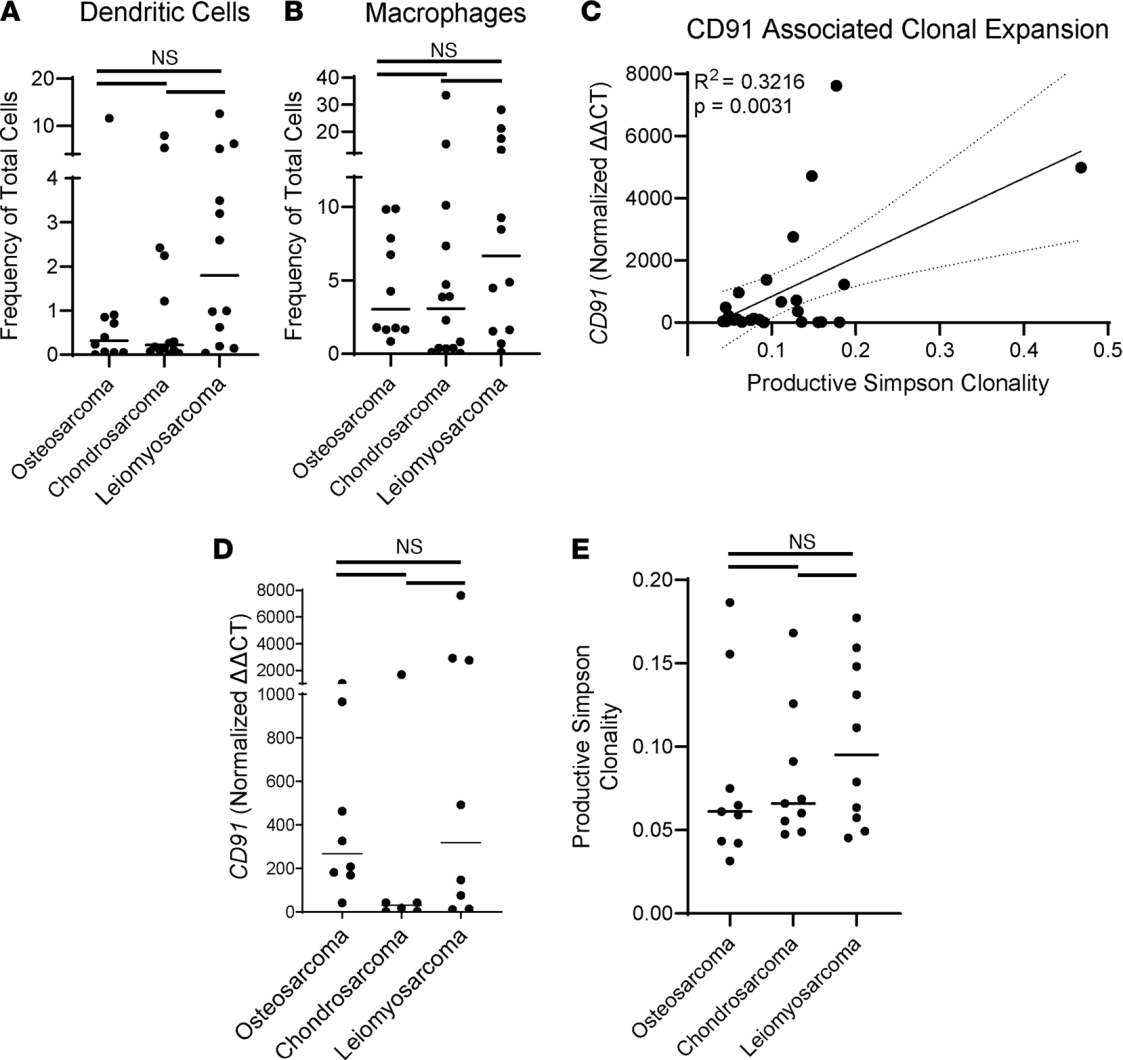
CD91 is one determinant for antitumor T cell responses in cancer immunosurveillance. The frequency of (**A**) dendritic cells (CD11b^+^CD11c^+^HLA-DR^+^) and (**B**) macrophages (CD3^–^CD56^–^CD11b^+^CD11c^–^CD33^+^) among total number of cells in the excised tumor was determined by flow cytometry for osteosarcomas (*n* = 10), chondrosarcomas (*n* = 14), and leiomyosarcomas (*n* = 12). (**C**) *CD91* expression within tumor samples from each patient was measured by qRT-PCR and plotted against PSC score (*n* = 25). (**D**) *CD91* mRNA expression and (**E**) PSC score are plotted for each tumor type (*n* = 9–10). Significance was determined by 1-way ANOVA (for **A**, **B**, **D**, and **E**) or simple linear regression (for **C**). **A**, **B**, **D**, and **E** are data points with mean bar.

Although the reason for poor immunosurveillance in patients with osteosarcoma is likely multifactorial, we examined some key facets for generation of tumor-specific T cell responses. Dendritic cells are key players for antigen cross-presentation to T cells and contribute to cancer immunosurveillance (36). DCs further provide costimulation and cytokines for T cell priming (40). We investigated DC percentages in tumors by flow cytometry and found relatively equal numbers of DCs in osteosarcomas, chondrosarcomas, and leiomyosarcomas (Figure 4A). There were also no differences in macrophages in these 3 tumor types (Figure 4B). Additional flow cytometry analyses on other sarcoma types in our patient cohort (for which whole-exome sequencing is unavailable) are available in Supplemental Figure 4. Our previous work showed that the HSP receptor, CD91, is a critical molecule expressed by DCs and macrophages that determines the competence of immunosurveillance in murine systems (8) and is associated with strong immune responses in patients with melanoma (28, 41). We investigated *CD91* expression in the tumors of patients by qRT-PCR and compared that with T cell responses as measured by PSC score. We observed a significant positive correlation of T cell responses to levels of *CD91* expressed (Figure 4C). When we dissected the data on osteosarcomas, chondrosarcomas, and leiomyosarcomas, we found no significant differences between these 3 sarcoma types in CD91 expression (Figure 4D) or PSC score (Figure 4E), indicating that although CD91 and T cell responses are both necessary for tumor-specific immunity, frequency and numbers do not appear to be leading factors in determining differences in immune responses to neo-epitopes expressed by these sarcomas (Figure 4E).

### HSP-mediated immune responses can reject osteosarcomas.

**Figure 5:**
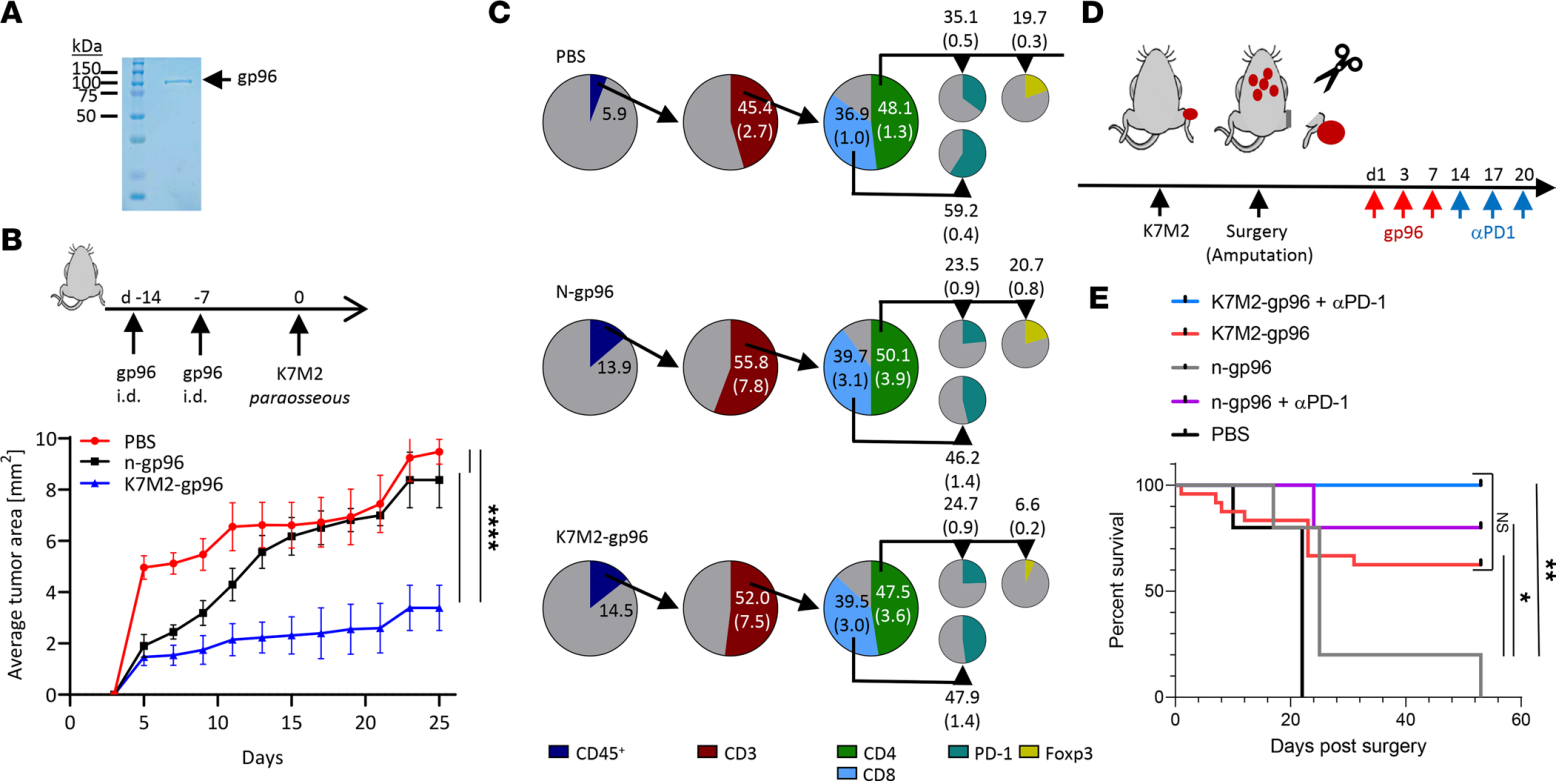
Gp96 primes T cell responses that mediate rejection of antigenic bone metastasis. (**A**) Tumor-derived gp96 was purified from K7M2 cells to apparent homogeneity as analyzed by SDS-PAGE. (**B**) Mice were immunized with PBS, normal tissue–derived gp96 (n-gp96), or tumor-derived gp96 (K7M2-gp96) twice, 7 days apart, and then challenged with K7M2 tumor cells, 7 days after final immunization, as shown in the schema. Tumor growth was monitored over time (*n* = 6–7). (**C**) Tumors from **B** were harvested 25 days after tumor challenge and analyzed by flow cytometry for immune cells (CD45^+^), then total T cells (CD3^+^), then T cell subsets (CD4^+^ and CD8^+^), and finally PD-1 expression (on CD4^+^ or CD8^+^ T cells) or FoxP3 (on CD4^+^ T cells). Frequency of the parent population is shown as well as the frequency of total cells in parentheses. (**D**) Mice were challenged with K7M2 paraosseously at the distal end of femurs, and tumors were allowed to grow to 0.7 to 1 cm in any diameter. The tumor-bearing limb was amputated and mice were randomized. Mice were treated 1 day later with PBS, n-gp96, or K7M2-gp96 alone or in conjunction with α–PD-1 as shown in the schema. (**E**) Survival of animals from **D** was assessed for 53 days (*n* = 20). Significance was determined by (**B**) ANOVA of area under the curve or (**E**) Kaplan-Meyer survival analysis. *P < 0.05, **P < 0.01, ****P < 0.0001.

Since osteosarcomas were found to express many highly antigenic neo-epitopes (Figure 2, D and J), we postulated that they should be particularly responsive to an immunotherapeutic regimen that concentrates on raising tumor-specific T cell responses. We therefore tested responsiveness of metastatic osteosarcoma in a mouse model. We used tumor-derived gp96 to prime T cell responses to K7M2, a mouse osteosarcoma, for the following reasons: (i) tumor-derived gp96 chaperones the antigenic repertoire of the tumor, including antigens that will constitute both high and low DAI neo-epitopes (42–44); (ii) HSPs prime particularly strong T cell responses because of their ability to cross-present their chaperoned peptides (29–32) and provide costimulation via DCs (33, 34) for those responses; (iii) ease of purification of HSPs (45); (iv) their ability to cause the rejection of tumors (35, 36); and (v) their demonstrated safety in the clinic (46–48). Gp96 was purified from K7M2 tumors (K7M2-gp96) to apparent homogeneity as determined by a single band by SDS-PAGE (Figure 5A). To demonstrate the ability of gp96 to prime tumor-specific immunity, BALB/c mice were immunized with 1 μg of K7M2-gp96 (or normal tissue–derived, n-gp96) twice as shown in Figure 5B’s schema, then challenged with syngeneic K7M2 tumor 1 week later. Tumor growth was measured and plotted as average tumor diameter (Figure 5B). Tumors in mice that were immunized with K7M2- gp96 grew significantly slower than tumors in mice immunized with n-gp96 or PBS. These data demonstrate that autologous, tumor-derived gp96 is capable of mounting tumor-specific immune responses, consistent with findings in many other tumor models (35–37). At the end of the experiment, on day 25, all remaining palpable tumors were harvested and immunophenotyped to determine the nature of the T cell response (Figure 5C). We found an increased immune infiltrate (CD45^+^) in tumors immunized with gp96 regardless of its source. This is consistent with the prior observations that gp96, regardless of its cellular source and independent of chaperoned tumor antigen, still mediates its adjuvanticity via CD91-expressing APCs (33, 34). Similar to the human disease (Figure 3C), murine osteosarcoma generated very poor T cell responses without therapeutic intervention (PBS group; Figure 5, B and C, top row). There was also an increased fraction of T cells (CD3) and T cell subsets in tumors immunized with gp96. In order to understand why tumors in the K7M2-gp96–immunized mice, but not n-gp96–immunized mice, were rejected, we analyzed T cells for expression of exhaustion markers such as programmed cell death 1 (PD-1), NKG2A, LAG3, TIM3, TOX, and TIGIT, but no differences were observed between the 2 groups. However, immunization with gp96 (regardless of source) significantly reduced PD-1 expression on both CD4^+^ and CD8^+^ T cells (Figure 5C). There were markedly fewer FoxP3^+^CD4^+^ cells in tumors from mice immunized with K7M2-gp96 compared with n-gp96–immunized or unimmunized mice (Figure 5C). The reduced number of Tregs is likely to contribute to the more effective T cell rejection of tumors. We proceeded to immunotherapy of these tumors with an HSP regimen (Figure 5D). We modeled the experiment to mimic the clinical situation of patients with osteosarcomas. K7M2 tumor cells, implanted paraosseously at the end of the femur to reflect the location of the overwhelming majority of osteosarcomas, were allowed to grow to 0.7–1.0 cm in diameter size, at which point metastatic lesions were established. Tumor-bearing limbs were amputated to rid the mice of primary tumors. Mice were randomized and treated with K7M2-gp96 or n-gp96 or left untreated. Given the high (almost 50% of the CD8^+^ T cells) expression of PD-1 in T cells primed with gp96 (Figure 5C), we included groups that received both gp96 and α–PD-1. α–PD-1 was given after gp96, allowing for the prior establishment of T cell responses and infiltration into tumors (Figure 5D). Mice were monitored for survival and assessed for metastases following death (Figure 5E). For monotherapy, K7M2-gp96 significantly prolonged survival of mice when compared with untreated mice or mice treated with n-gp96. In the dual-therapy setup, additional treatment of mice with α–PD-1 following K7M2-gp96 extended survival to 100%. Metastatic K7M2 has previously been shown to be unresponsive to α–programmed cell death ligand 1 therapy (49). Interestingly, the addition of α–PD-1 to mice treated with n-gp96 showed significant benefit over treatment with n-gp96 alone (Figure 5E); however, this was not as effective as K7M2-gp96 + α–PD-1, where we observed 100% survival. All mice that succumbed to disease had extensive lung metastases, regardless of treatment group. At the conclusion of the experiment, all remaining mice were sacrificed, and no gross metastatic burden was observed. These studies demonstrate the sensitivity of osteosarcoma-derived metastatic lesions to T cell–mediated immunotherapy and a potentially novel immunotherapy regimen that could be applicable in the clinic.

## Discussion

Due to their relatively low prevalence compared with tumors of other origins (38), the immunology of sarcomas has been understudied, and therapeutic options have remained stagnant for the last 30 years (50). Indeed, the excitement surrounding checkpoint blockade therapy for several carcinomas has not translated to sarcomas (51, 52) largely because T cell responses, a requirement for immune checkpoint blockade (ICB) therapy, and the antigenicity of these tumors are poorly understood. Measuring antigenicity and immunogenicity of mouse tumors is typically done directly through transplantation studies (3, 4) and, for years, has been used to identify tumor-specific peptides that are processed and presented by MHC to generate tumor-rejecting T cell responses (53). Without a similar approach for human tumors, it has proved difficult to measure antigenicity of human tumors. In our study we utilized a newly developed index to determine the antigenic landscape of sarcomas freshly isolated from patients (8, 14, 23). When DAI was used to classify neo-epitopes, we were able to distinguish the fine differences in sarcoma antigenicity. Osteosarcomas in particular have many high-DAI epitopes, potentially making them conducive targets for T cell–based immunotherapy. Leiomyosarcomas and liposarcomas appeared to be devoid of such high-DAI neo-epitopes. These differences were not observable using the CDN definition of neo-epitopes, providing further impetus for the utility of DAI classification for neo-epitopes. This is important for selection of neo-epitopes that are developed for immunotherapy of cancer.

Genomic analysis of the TCRβ repertoire, as opposed to a simple measure of immune infiltrate at time of surgery, offers insight into antitumor T cell responses, including memory responses, and a measure of the impact of immunosurveillance pressures on the emergent tumor (54). This likely reflects tumor emergence, the duration of the equilibrium state, and the progression (escape) of the tumors (55). For the first time to our knowledge, we have combined this metric with the DAI analysis to establish a correlation wherein patients with strong T cell responses have shaped their tumors such that the tumors no longer express dominant antigens that can be targeted by the T cells and vice versa. This is evidence of longitudinal and continuous immunosurveillance of tumors and may be applied to other forms of malignancies. In this cohort of patients, the CD4^+^ and CD8^+^ T cell infiltrates did not correlate with the PSC score. As T cell infiltration certainly varies per tumor type, a measure of TIL at time of surgery may offer little/no insight into prior antitumor reactions and responses, especially for advanced tumors.

An unanswered question is why patients with osteosarcomas mount poor T cell immune responses. Previous studies have shown that these tumors have genomic instability and are predicted to have many new mutations (22, 56, 57). However, our study shows that the number of SNVs was not significantly higher than other sarcomas, and indeed, neither was the total number of neo-epitopes defined by DAI or CDN. Although high-DAI neo-epitopes are expressed on these tumors, they are not being reactive. This is a classical definition of T cell ignorance, which can be broken by immunization (in this case with HSP-peptide complexes). The answer is probably multifactorial. One of the key molecules necessary for the cross-priming of neo-epitopes is the HSP receptor, CD91 (8). In mouse models of cancer, CD91 is indispensable for cancer immunosurveillance. Functionally, CD91 is important for driving cancer-associated costimulation, cytokines, and cross-presentation that are necessary for priming antitumor T cell responses (29–34). Correlation of CD91 expression with clinical benefit has emerged in patients with melanoma (28). Consistent with these studies, here we observed a strong correlation between CD91 expression and T cell responses overall, likely indicating its necessity in priming antitumor immune responses. When tumors are large and antigen burden is significantly increased, other mechanisms of antigen capture may also be relevant (58, 59). However, we did not observe differences in CD91 expression between patients with osteosarcoma compared with patients with leiomyosarcomas or liposarcomas, which would have provided a plausible explanation for differences in T cell responses in these patients. Although CD91 expression does correlate with the frequency of DCs found within these tumors (Supplemental Figure 5), we cannot confirm the identity of all cell types that express CD91 within the tumor microenvironment. Several HSPs have been confirmed as ligands for CD91, and the HSP-CD91 engagement leads to robust antitumor immunity (8, 29, 30, 35, 36). As we show here, CD91 expression is variable in humans, and while low CD91 expression correlates with worse prognosis in cancer, it is conceivable, though unlikely, that ectopic and excessive CD91 expression by tumor cells themselves could be immunosuppressive by acting as an HSP sink, limiting uptake by APCs. There was also no observable difference in intratumor APC numbers at time of tumor resection, although this does not eliminate the possibility of differences at time of initial priming of T cell responses or within the tumor-draining lymph nodes. Allelic loss or β2m mutations, leading to downregulation of MHC, may also account for poorer T cell responses in some patients and accumulation of tumors with high-DAI neo-epitopes.

Chemotherapy and radiation, prior to surgery, could have effects on measurements of immune parameters. For the limited number of patients who received chemotherapy or radiation prior to surgery, as much as was practically possible, samples were obtained at minimum 2 weeks but mostly many years removed from this presurgery regimen to minimize potential artifacts on immune cells (Supplemental Table 1). Subsequently, our analysis did not reveal effects of chemotherapy on TILs. We are excited about the possibility of modeling this in mouse tumors. Given the expression of many neo-epitopes with high DAI in osteosarcoma, we selected this tumor type for experimentation in mice. We first established that tumor-derived gp96 mounted beneficial T cell responses in mice following immunization. This is an observation consistent with many other murine tumor types (31, 35, 36, 42, 43). We found that gp96, regardless of its source (tumor or normal tissue), was able to induce immune and T cell infiltration into tumors, which we attribute to its inherent adjuvanticity (33, 34). However, tumor rejection was only achieved when gp96 was derived from tumor (and thus chaperoning tumor antigens). Our data here emphasize the importance of antigen specificity in generating long-term, sustainable responses that are also therapeutically beneficial. When mice bearing osteosarcoma-derived lung metastasis were treated with tumor-derived gp96, mice survived markedly longer than controls. Since tumor-derived gp96 confers its antitumor immune responses by priming effector CD4^+^ and CD8^+^ T cells, we demonstrated that this can be augmented with ICB. This is an important finding given that ICB for osteosarcoma, without consideration of preexisting T cell response, has not yielded clinical benefit (50–52). Interestingly, n-gp96 (derived from normal tissue) provided some survival benefit when combined with α–PD-1, but not alone. Our previous findings have shown that gp96, regardless of its source (or bound antigen), is able to provide adjuvanticity for antitumor responses (33, 34). While this may be insufficient by itself (without the antigen-specific component) to extend survival in our study, α–PD-1 is able to synergize with this adjuvanticity to provide some therapeutic benefit. This is further enhanced when tumor-derived gp96 is used with α–PD-1, thereby providing the antigen-specific immune responses.

With a systematic analysis of neo-epitopes on sarcomas, we demonstrate antigenic differences in these tumors and show this is a result of active immunosurveillance of tumors, with T cell–mediated immune responses clearing tumors expressing immunogenic neo-epitopes. Further work is needed to elucidate why patients with osteosarcomas mount poor T cell responses. However, these tumors emerge with conducive immunogenic neo-epitopes, which we demonstrate are particularly amenable to T cell–based immunotherapy.

## Methods

### Tumor and blood tissue.

Tumors were primary, metastatic, or recurrent. Broad treatment regimens and demographics for patients are provided in Tables 1–3 and Supplemental Table 1. Funding for the registry is in Acknowledgments.

### Prediction of neo-epitopes.

Whole-exome sequences from blood samples were used to determine 4-digit HLA-I haplotypes using Seq2HLA (Supplemental Table 2). Tumor mutations were identified using the consensus caller cross-platform, comparing fastq data from tumor (Mut) and blood (WT) whole-exome sequencing. Prediction of neo-epitopes was performed based on Consensus CDS annotations using a custom Galaxy tool. For each nonsynonymous somatic mutation, we used NetMHC 4.0 to compute the predicted binding affinity (IC_50_) of each 8-, 9-, 10-, and 11-mer–mutated peptide. IC_50_ values were scored using the profile weight matrix algorithm. DAI was calculated by subtracting the WT score from the Mut score. This was done for each patient’s identified HLA-A, -B, and -C alleles.

### Exome sequencing.

Genomic DNA from tumors and blood was purified using the QIAGEN Puregene Kit (catalog 158445). DNA from buffy coats was purified using QIAGEN DNeasy Blood & Tissue Kit (catalog 69504). The Genomic DNA was sheared with Ion Shear Plus Reagents (Ion Plus Fragment Library Kit) and size-selected with Agencourt AMPure XP beads (Beckman Coulter). DNA fragments with a base pair peak of 100–150 bp were ligated with Ion adapters, purified with Agencourt AMPure XP beads, and PCR-amplified. Then, 750 ng of the adapter-ligated DNA library was hybridized to SureSelect capture library (Agilent SureSelect XT Mouse All Exon Kit) for 20 hours at 65°C. The hybrid capture library was selected using Dynabeads MyOne Streptavidin T1 beads (Life Technologies). The captured library was amplified and purified with AMPureXP beads, and quality was assessed on the High Sensitivity DNA Kit (Life Technologies) on the Agilent Bioanalyzer. We selected a 220 bp peak using E-Gel SizeSelect 2% agarose gel (Life Technologies). The final library was purified and quality assessed on High Sensitivity DNA Bioanalyzer chip (Life Technologies). Templates were prepared using the Ion PI Hi-Q Chef Kit (Life Technologies) on the Ion Chef platform and sequenced on an Ion Proton Sequencer on a PI v3 chip using Ion PI Hi-Q Sequencing 200 Kit (Life Technologies).

### TCR sequencing.

Genomic DNA was isolated from tumor tissue using the QIAGEN Puregene Kit, and the CDR3 variable region of *TCRB* was amplified by PCR using the immunoSEQ Human TCRB kit (Adaptive Biotechnologies). Amplified products were sequenced by the Genomics Research Core at the University of Pittsburgh. Sequences were uploaded to the immunoSEQ analysis platform (Adaptive Biotechnologies) and used to determine PSC score. Simpson clonality is calculated as the square root of Simpson’s diversity index (a measure of diversity within a population, which takes into account both the number and abundance of different members within that population). It results in a value from 0 to 1, where 0 indicates a population of entirely unique TCRs and 1 is a homogeneous population of a single TCR.

To calculate antigen-associated clonal expansion, for each patient, the average number of epitopes within set DAI ranges was determined (i.e., ≥9, 8–8.99, 7–7.99, etc.). The number of epitopes was normalized within each range, with the maximum value being 100. The normalized values in each range were plotted against the corresponding PSC. The slope and error of the linear regression line from each of these comparisons were calculated as the antigen-associated clonal expansion value.

### qRT-PCR.

RNA was extracted from less than 30 mg of tumor tissue using QIAGEN RNeasy Mini Kit. The extracted RNA was converted to cDNA, priming with oligo(dT) following the SuperScript III First-Strand Synthesis System for RT-PCR (Invitrogen). cDNA obtained was used to measure the expression levels of *CD91* using *GAPDH* for normalization. Multiple primer pairs spanning the α and β subunits of *CD91* were used. For each patient, samples were normalized to their own *GAPDH* expression, then normalized to a single reference sample to yield the ΔΔCt value.

### Mice and tumor cell line.

Female (WT) BALB/c mice (strain 000651) were purchased from The Jackson Laboratory. Mice were housed in the animal facility Division of Laboratory Animal Resources at the University of Pittsburgh (Pittsburgh, Pennsylvania, USA). K7M2 cells were obtained from ATCC and cultured as recommended, in complete DMEM (10% FBS). Cells were harvested with trypsin, washed with PBS, and suspended in PBS prior to use in vivo.

### Tumor prophylaxis and therapy experiments.

For prophylaxis experiments, mice were immunized twice each with 1 μg of gp96 in 100 μL saline, intradermally 1 week apart. Mice were challenged with 500,000 K7M2 intradermally 1 week later. Tumor growth was monitored by measurement of the tumor on 2 axes with calipers. The average tumor diameter was calculated. For the therapy experiments, mice were challenged with 500,000 K7M2 tumor cells paraosseously at the ends of the right femur. When primary tumors were 0.7–1 cm in any diameter, mice were anesthetized and affected limbs were amputated (to mimic radical osteosarcoma treatment protocol) with a sterile scalpel blade after ligation of the femoral vascular bundle. Skin was closed in a tension-free manner. Mice received analgesia and were monitored daily following surgery. Mice were randomized and placed in groups that received 3 doses of 1 μg of gp96 in 100 μL or 100 μL PBS 2 days apart, starting on day 1 after surgery. Some groups (as indicated in Figure 5) received 3 additional doses of 200 μg of α–PD-1 (catalog BP0273, Bio X Cell) every 3 days intraperitoneally. Mice were monitored for survival. All dead mice had the appearance of pulmonary metastases.

### Purification of gp96.

Gp96 was purified from healthy murine livers or from K7M2 tumors harvested from mice to obtain normal (n-gp96) or tumor-derived (K7M2-gp96), respectively. Detailed procedures have been previously described (45). All purified gp96 preparations were verified by SDS-PAGE to be homogenous (see Figure 5A as an example) and routinely immunoblotted with gp96-specific antibodies. As determined by Limulus amebocyte lysate assay, preparations had <0.001 endotoxin units/μg of endotoxin. Gp96 was concentrated to 1 mg/mL, aliquoted, and stored at –80°C until use.

### Flow cytometry.

Fresh human tumor tissue or K7M2 mouse tumors were mechanically disrupted, followed by enzymatic digestion using 0.2% collagenase D (MilliporeSigma) with 2% BSA in RPMI (MilliporeSigma). Cells were resuspended in PBS for cell surface antibody staining, then fixed and permeabilized with Fix & Perm solution for intracellular staining (5523-00, Thermo Fisher Scientific). Cells were resuspended in 1% BSA and 0.1% sodium in 1× PBS and filtered for flow cytometry data acquisition. Data were acquired with BD Biosciences LSR II and analyzed using FlowJo software (Tree Star Inc.). The following anti-human antibodies were used: Fc block (catalog 564220), CD45 (catalog 563792), CD4 (catalog 557852), CD33 (catalog 561160), CD3 (catalog 564307), HLA-DR (catalog 562845), CD8 (catalog 557834), CD25 (catalog 560503), CD11b (catalog 555389), CD11c (catalog 559877), and CD56 (catalog 560360), all from BD Biosciences. The following anti-mouse antibodies were used: Fc block (catalog 564220), Zombie (catalog 745697), CD45 (catalog 563053), CD4 (catalog 612843), CD8 (catalog 560778), and CD3e (catalog 555278) from BD Biosciences; LAG3 (catalog 48-2231-82), TIGIT (catalog 46-9501-82), and FoxP3 (catalog 17-5773-80B) from Thermo Fisher Scientific; NKG2A (FAB6867N-1) from BioLegend; PD-1 (catalog 109116-127) from Jackson ImmunoResearch; TOX (catalog 130-120-785) from Labome; and 2B4 (catalog ab95806) from Abcam.

### Statistics.

Statistical analyses were performed with GraphPad Prism Software version 9 using 1-way ANOVA for comparison between 2 variables. Statistical significance was defined as *P* < 0.05.

### Study approval.

All samples were obtained from consenting patients enrolled in the MOTOR (STUDY20010034), established in 2012, at the University of Pittsburgh Department of Orthopaedic Surgery and Hillman Cancer Center. This research registry provided deidentified tumor tissue and blood samples for the study.

All experimental mice were 6 to 8 weeks old. All experiments with mice were approved by the Institutional Animal Care and Use Committee at the University of Pittsburgh and performed in compliance with its guidelines.

### Data availability.

All underlying data are available from the corresponding author upon request. Anonymized human participant data are provided in Supplemental Tables 1 and 2. The whole-exome data are publicly accessible at Sequence Read Archive at NIH (accession number PRJNA987736).

## Author contributions

DOH, ALS, LMH, AAY, SGI, KRW, and RJB performed experiments in this manuscript. RJB conceived the project. RJB wrote the manuscript with input from all contributing authors. DOH and KRW additionally provided access to the human samples. Co–first authors DOH and ALS are listed in alphabetical order.

## Supplementary Material

Supplemental data

## Figures and Tables

**Table 1:**
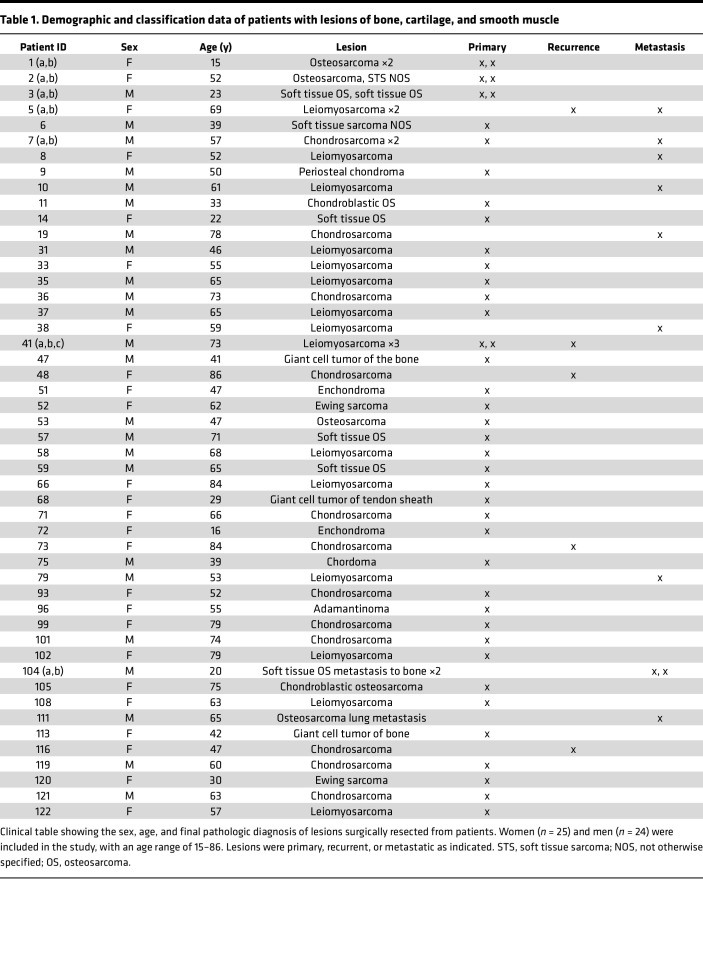
Demographic and classification data of patients with lesions of bone, cartilage, and smooth muscle

**Table 2:**
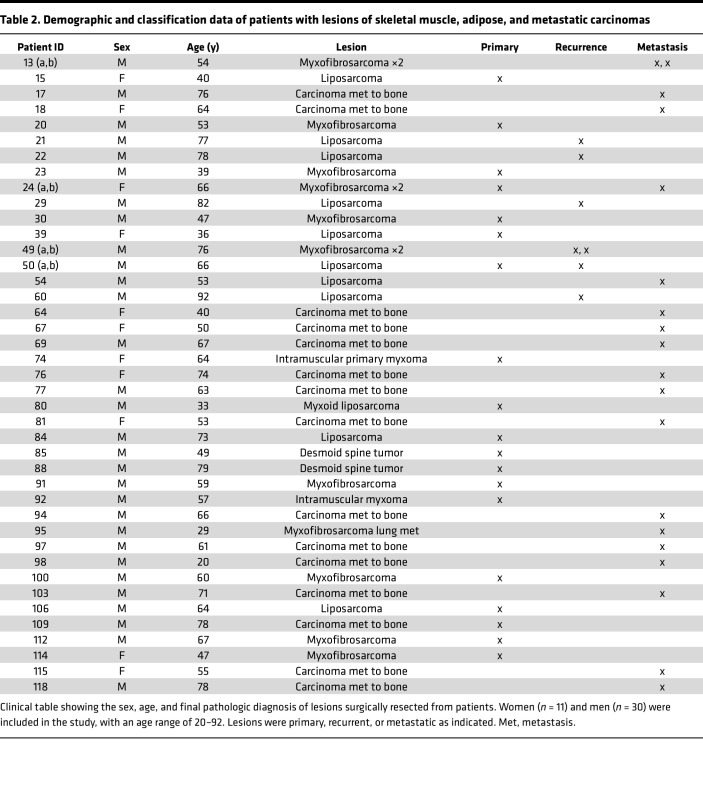
Demographic and classification data of patients with lesions of skeletal muscle, adipose, and metastatic carcinomas

**Table 3:**
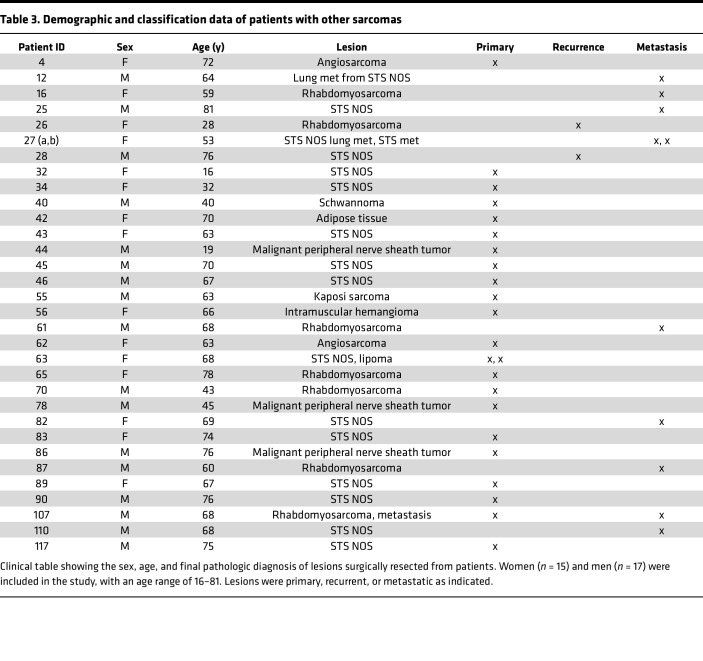
Demographic and classification data of patients with other sarcomas

## Version 1 07/10/2023

Electronic publication

## Version 2 07/12/2023

David Osei-Hwedieh's present address was added
