# Supplementary material for: Immunosurveillance shapes the emergence of neo-epitope landscapes of sarcomas, revealing prime targets for immunotherapy
Source: JCI Insight. 2023 Jul 10;8(13):e170324. doi: 10.1172/jci.insight.170324 (PMC10371341; doi:10.1172/jci.insight.170324)
Supplement: Supplemental data [file jciinsight-8-170324-s252.pdf]

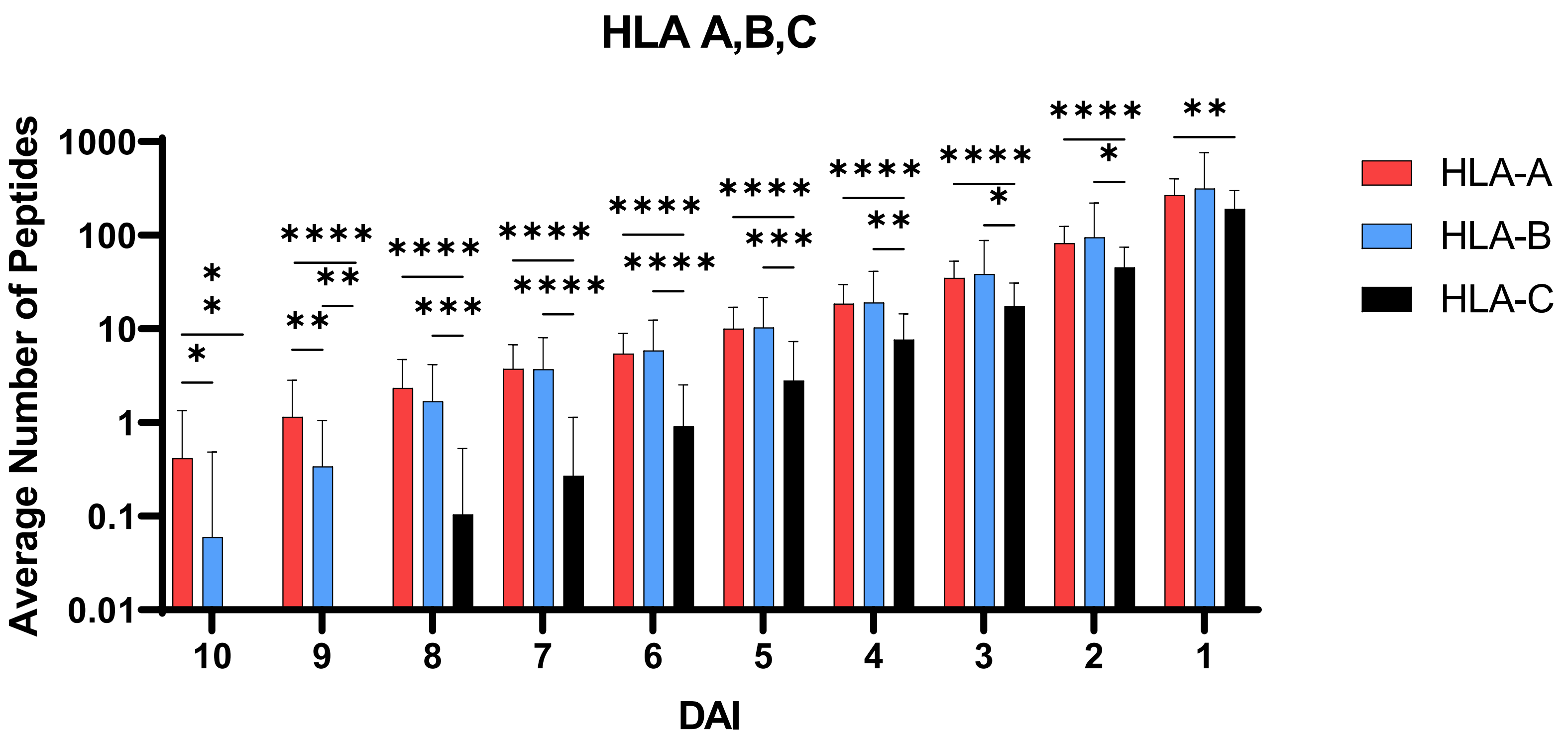

Supplementary Figure 1. HLA class 1 may influence immunosurveillance. The average number of mutated peptides with varying DAI presented by HLA class 1 molecules is presented. *p<0.05, **p<0.01, ***p<0.005, ****p < 0.0001.

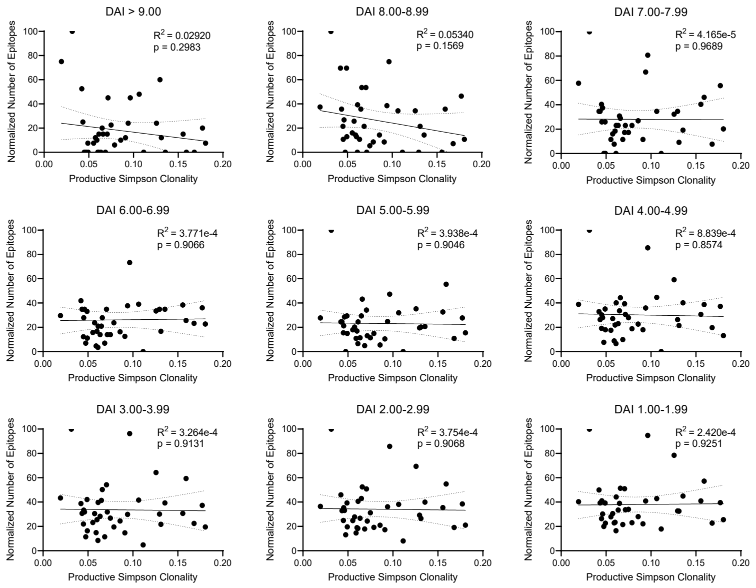

Supp Fig 2. Negative correlation between number of epitopes and PSC only exists at high DAI values. The average number of epitopes for each DAI range was normalized to a scale with max 100. These values were plotted against PSC and linear regression analysis was performed.

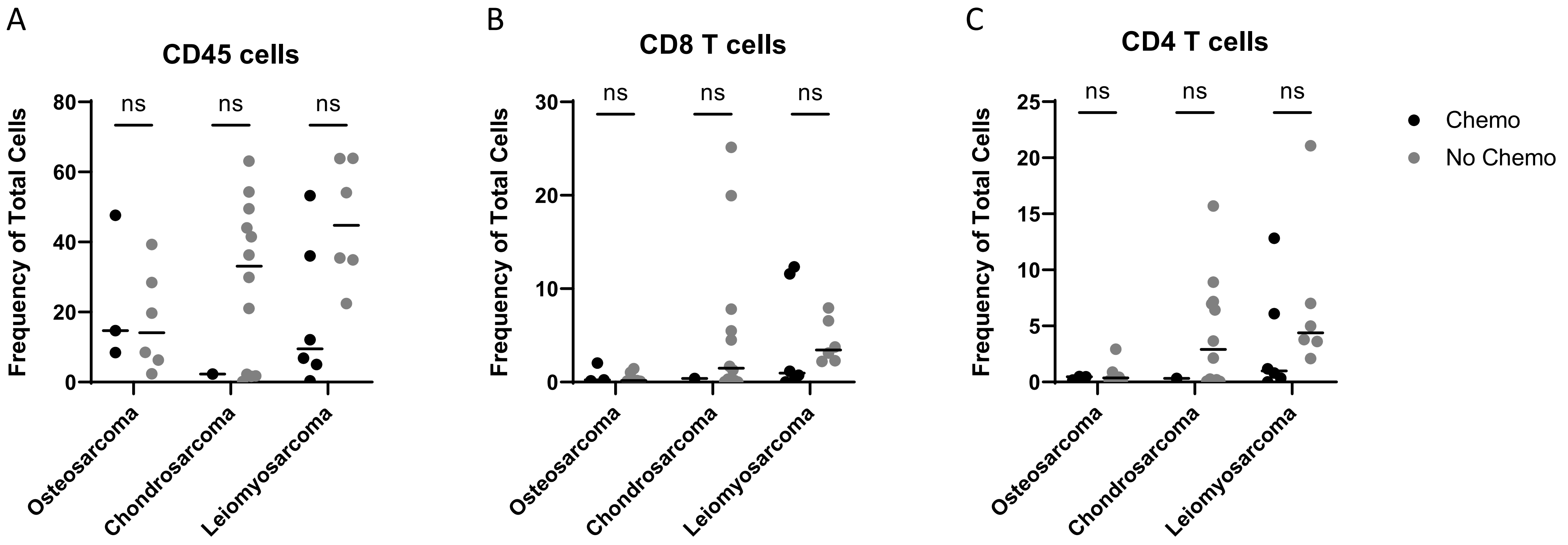

Supplementary Figure 3. Prior treatment with chemotherapy did not significantly alter patient immune or T cell infiltrate. Flow cytometry of $CD45^+$, $CD8^+$ or $CD4^+$ cells were analyzed from patients stratified based on whether they received chemotherapy prior to surgery to harvest tumors. Significance was determined by two-way ANOVA with Sidak's multiple comparisons test. ns is not significant.

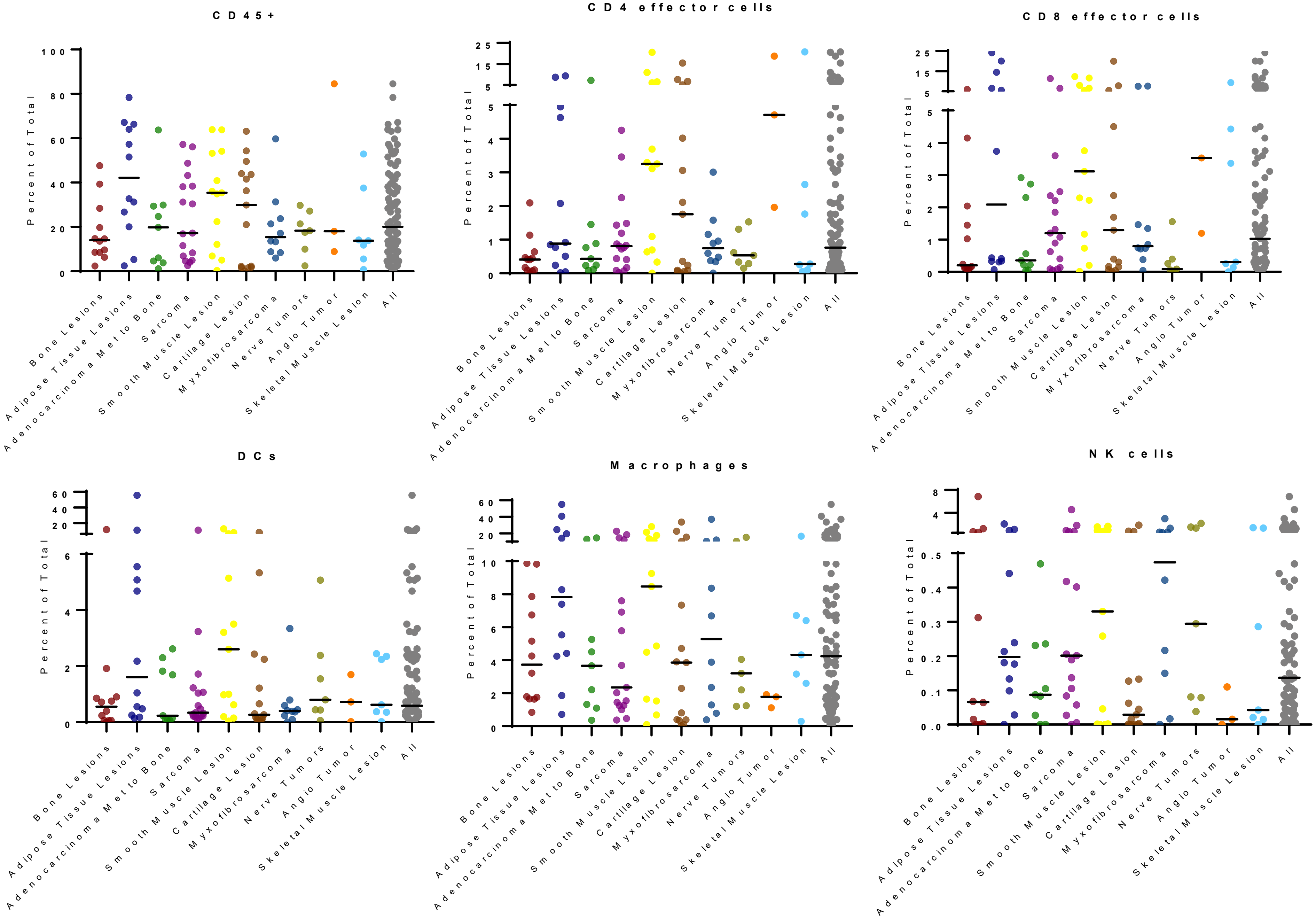

Supplemetary Figure 4. **Immune cell populations within sarcomas.** Tumor infiltrating immune cells were analyzed by flow cytometry on excised tumor samples from 122 patients. The classification of tumors is in Table 1 and Supplementary Table 1, which has additional demographics. Bar in each column (tumor type) represents the mean of that column.

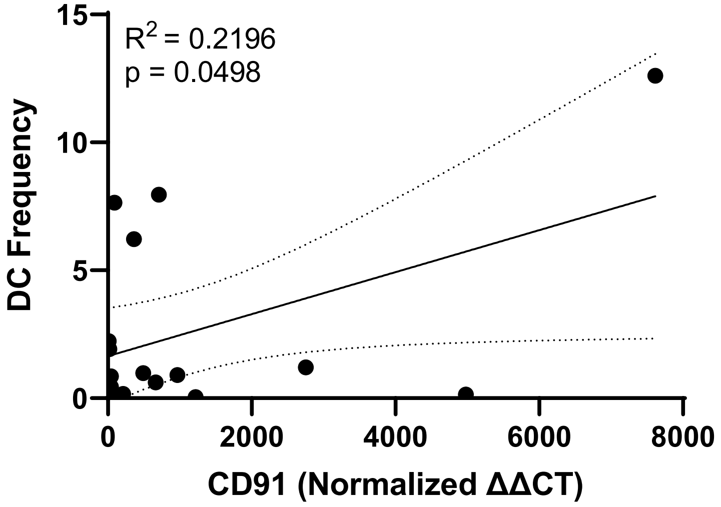

Supplementary Figure 5. Intratumoral expression of CD91 correlates with DC infiltration. The frequency of DCs among total cells in the tumor was plotted against CD91 mRNA expression. Significance was determined by linear regression.

Patient ID	Sarcoma subtype	Chemotherapy prior to surgery	Day of last chemotherapy before Surgery	Radiation therapy prior to surgery	Day of last radiation therapy before Surgery
1a	Bone	no	-	no	-
1b	Osteosarcoma, high grade, primary	yes	22	no	-
2a	Osteosarcoma, radiation induced	yes	> 10 yrs	yes	> 10 yrs
2b	high grade radiation induced sarcoma, primary	yes	> 10 yrs	yes	> 10 yrs
3a	Soft tissue osteosarcoma	yes	14	no	-
3b		yes	48	no	-
4	Scalp angiosarcoma	no	-	no	-
5a	recurrent high grade leiomyosarcoma	no	-	yes	192
5b	metastatic high-grade leiomyosarcoma (recurrence v metastasis, in same limb as primary but seems to be migrating up the arm)	yes	87	yes	412
6	Giant cell reaction suture granulomae/no residual sarcoma tumor identified	no	-	no	-
7a	Intermediate grade chondrosarcoma	no	-	no	-
7b	metastatic chondrosarcoma	no	-	no	-
8	metastatic high grade leiomyosarcoma	yes	39	no	-
9	periosteal chondroma	no	-	no	-
10	metastatic leiomyosarcoma, high grade	no	-	no	
11	chondroblastic osteosarcoma, primary	no	-	no	-
12	Pulm met sarcoma w/ hyalinization & necrosis	yes	28	yes	369
13a	metastatic myxofibrosarcoma	no	-	yes	405
13b	metastatic myxofibrosarcoma	no	-	-	-
14	Tibial high grade osteosarcoma	yes	39	no	-
15	Myxoid liposarcoma	no	-	no	
16	Metastatic pleomorphic rhabdomyosarcoma	no	-	no	-
17	malignant melanoma, Nodular and spindle cell type	no	-	yes	578
18	Metastatic squamous cell carcinoma	yes	1082	-	-
19	Dediff chondrosarcoma with rhabdomyosarcomatous	no	-	Yes	> 10 yrs

	and osteosarcomatous components, high grade, primary				
20	myxofibrosarcoma, intermediate grade	yes	554	yes	864
21	Recurrent dediff liposarcoma with osseous metaplasia and osteoclast-like giant cell rich areas (polymorphic)	yes	960	-	-
22	recurrent well-differentiated liposarcoma	no	-	no	-
23	Fibrotic tissue with scant myxoid stroma, suspicious of microscopic residual sarcoma	no	-	no	-
24a	High grade myxofibrosarcoma	no	-	no	-
24b	Metastatic high grade myxofibrosarcoma (this is lung tissue, original tumor in R thigh)	no	-	Yes	132
25	metastatic high grade sarcoma	no	-	-	-
26	rhabdomyosarcoma, high-grade, recurrence	yes	225	no	-
27a	metastatic sarcoma, three foci consistent with prior hx of undifferentiated pleomorphic sarcoma, lung met	yes	30	-	-
27b	metastatic pleomorphic sarcoma (4.3 cm)	yes	178	-	-
28	Spindle cell sarcoma, intermediate grade, recurrence ("non-classifiable")	no	-	-	
29	recurrent dedifferentiated liposarcoma, high grade	yes	N/A	yes	45
30	Benign fibroma (suspected Myxofibrosarcoma) - negative for malignancy, however felt to be sampling error, hx of widely metastatic myxofibrosarcoma	yes	78	-	-
31	Angioleiomyoma	no	-	yes	37
32	Biphasic synovial sarcoma with extensive postchemotherapy necrosis and cystic degeneration	yes	75	yes	50+ years
33	Leiomyosarcoma, high grade	yes	> 50 yrs	-	-
34	Residual Monophasic Synovial Sarcoma	no	-	yes	141

	involving dermis, subcutaneous and fascia				
35	favor leiomyosarcoma, high grade, post-radiation, not considered metastatic	yes	143	-	-
36	dedifferentiated chondrosarcoma	no	-	no	-
37	high grade Leiomyosarcoma- Sigmoid colon	no	-	no	-
38	Metastatic High grade Leiomyosarcoma	yes	1351	no	-
39	Dedifferentiated Liposarcoma	no	-	no	-
40	Schwannoma with organizing thrombus and papillary endothelial hyperplasia (Masson's change)	n/a	-	n/a	-
41a	primary high grade leiomyosarcoma	no	-	no	-
41b	primary high grade leiomyosarcoma	no	-	no	-
41c	Recurrent high grade Leiomyosarcoma -Thigh	no	-	no	-
42	Benign fibroadipose tissue with seroma cavity	no	-	no	-
43	Intermediate grade spindle cell sarcoma, not further specified.	no	-	no	-
44	Neurofibromatosis with few small foci of malignant PNST	no	-	no	-
45	high grade sarcoma, 99% therapy effect	yes	42	no	
46	No viable tumor found, hx of high grade sarcoma	yes	32	yes	2057
47	Giant cell tumor of bone with secondary aneurysmal bone cyst.	n/a	-	yes	527
48	recurrent chondrosarcoma	no	-	no	-
49a	recurrent myxofibrosarcoma, high grade	no	-	yes	1913
49b	recurrent myxofibrosarcoma, high grade	no	-	yes	2057
50a	recurrent dedifferentiated liposarcoma, high grade	yes	391	yes	527
50b	primary dedifferentiated liposarcoma, high grade	no	-	no	-
51	enchondroma	no	-	no	
52	Ewing sarcoma	no	-	no	
53	osteosarcoma, low to intermediate grade with	n/a	-	n/a	-

	invasion of medullary cavity - primary				
54	Metastatic pleomorphic liposarcoma, high grade	yes	310	no	-
55	Kaposi sarcoma in toe mass but not calf mass	n/a	-	n/a	-
56	Intramuscular hemangioma	n/a	-	yes	57
57	Extraskeletal osteosarcoma, intermediate to high-grade, involving soft tissue (10.2 cm)	n/a	-	n/a	-
58	primary leiomyosarcoma, high grade, involving mesorectum and mesentery of distal sigmoid w/o colonic invasion (12 cm)	no	-	no	-
59	extraskeletal osteosarcoma, high grade (19.2 cm)	no	-	no	-
60	Recurrent dedifferentiated liposarcoma, high grade	no	-	no	-
61	metastatic high grade sarcoma with myogenic differentiation most suggestive of sclerosing/spindle cell rhabdomyosarcoma.	no	-	no	-
62	multifocal high grade epithelioid angiosarcomaa - thigh	yes	39	yes	57
63	necrotizing granulomatous inflammation most suggestive of pseudotumor-Thigh	no	-	yes	329
	Pseudotumor (benign fibroadipose tissue) - Femur	no	-	no	-
64	Breast adenocarcinoma metastasis to femur	n/a	-	n/a	-
65	high-grade sarcoma with myogenic differentiation - thigh	no	-	no	-
66	leiomyosarcoma, intermediate grade - thigh	no	-	no	-
67	Breast adenocarcinoma met to femur	n/a	-	n/a	-
68	Giant cell tumor of tendon sheath - Ankle	n/a	-	n/a	-
69	Lung adenocarcinoma met to Femur	n/a	-	n/a	-
70	high grade pleomorphic myogenic sarcoma most	no	-	no	-

	compatible with pleomorphic rhabdomyosarcoma				
71	low grade (grade 1) chondrosarcoma	no	-	no	-
72	Enchondroma	n/a	-	n/a	-
73	De-differentiated Chondrosarcoma	no	-	yes	329
74	intramuscular primary myxoma	no	-	yes	43
75	primary chordoma - sacrum	no	-	no	-
76	Breast adenocarcinoma metastasis to acetabulum	n/a	-	n/a	-
77	Metastatic carcinoma with squamous differentiation, favoring a squamous cell carcinoma from L acetabulum	n/a	-	n/a	-
78	primary MPNST nerve sheath tumor	no	-	no	-
79	Metastatic (uterine) leiomyosarcoma sarcoma (sample is a lung met, primary was uterine)	yes	38	no	-
80	Myxoid liposarcoma from thigh	yes	57	no	-
81	Lung adenocarcinoma metastasis, poorly differentiated to humerus	n/a	-	n/a	-
82	Metastatic Fibroblastic Reticular Cell sarcoma from lymph node	no	-	-	-
83	Primary undifferentiated pleomorphic sarcoma, high grade (sacrum)	yes	41	no	-
84	Primary dedifferentiated liposarcoma from thigh	no	-	yes	862
85	Desmoid tumor from spine	n/a	-	n/a	-
86	primary MPNST pelvis	no	-	no	-
87	metastatic high grade pleomorphic rhabdomyosarcoma	yes	63	-	-
88	Desmoid tumor	n/a	-	n/a	-
89	No evidence of overt malignancy-overall features suggest a traumatic / infectious process with surrounding, exuberant reactive change and ischemic fascitis	n/a	-	n/a	-
90	Synovial chondromatosis - Right shoulder	n/a	-	n/a	-

91	high grade sarcoma c/w myxofibrosarcoma - right knee	no	-	no	-
92	intramuscular myxoma - thigh	no	-	no	-
93	primary conventional chondrosarcoma, grade 2 with focal grade 3 (area given for research was grade 2 - surrounding coxa)	no	-	yes	453
94	History of breast cancer (possible metastasis)	n/a	-	n/a	-
95	Lung Met – Myxofibrosarcoma	yes	63	-	-
96	Adamantinoma - Tibia	no	-	no	-
97	Metastatic Breast cancer- Left humerus	n/a	-	n/a	-
98	Plasma cell neoplasm metastasis to bone	no	-	no	
99	Possible Chondrosarcoma - pelvis	yes	> 10 yrs	yes	> 10 yrs
100	Myxofibrosarcoma- Thigh	no	-	no	--
101	Extraskeletal myxoid chondrosarcoma- Inguinal region	no	-	yes	862
102	High grade sarcoma c/w leiomyosarcoma	no	-	no	-
103	Metastatic Breast cancer - Acetabulum	n/a	-	n/a	-
104a	Metastatic Osteosarcoma- Femur	yes	912	no	-
104b	Metastatic Osteosarcoma- Tibia	yes	912	no	-
105	Chondroblastic osteosarcoma- Right hemipelvectomy	n/a	-	n/a	-
106	Liposarcoma	no	-	no	-
107	Rhabdomyosarcoma	yes	479	yes	795
	Rhabdomyosarcoma Lung metastasis. Spindle cell sclerosing type rhabdomyosarcoma	yes	532	yes	848
108	Pelvic leiomyosarcoma	no	-	no	-
109	Spindle cell recurrence metastasis to bone	no	-	yes	n/a
110	Sarcoma NOS	no	-	no	--
111	Osteosarcoma Lung metastasis	yes	39	yes	453
112	Myxofibrosarcoma	n/a	-	n/a	-
113	Giant Cell tumor of the bone	n/a		n/a	-
114	Malignant Myxoid epitheloid tumor	no	-	no	-

115	Metastatic cancer to the humerus	n/a	-	n/a	-
116	revealing recurrent dediff chondrosarcoma	yes	255	no	-
117	Soft tissue sarcoma from thigh (NOS)	no	-	no	-
118	Nodular and spindle cell type melanoma	no	-	no	-
119	Chondrosarcoma from femur	n/a	-	n/a	-
120	Ewing sarcoma	n/a	-	n/a	
121	Chondrosarcoma	n/a	-	n/a	-
122	Leiomyosarcoma	n/a	-	n/a	-

Supplementary Table 1. Therapeutic regimen of patients with sarcoma. Clinical table showing the therapy that each patient received and length of time prior to surgery and specimen collection. Lesions were with primary, recurrent or metastatic as indicated.

Patient Number	Confirmed HLA					
	A alleles		B alleles		C alleles	
1	A*03:01	A*29:01	B*44:03/26	B*15:01	C*16:01	C*03:04
2	A*03:01	A*02:01/22	B*40:01/10	B*14:02	C*03:04	am
3	A*30:04	am	B*07:02	am	C*17:01	C*07:02
5	am	am	B*44:02/26	B*15:04	C*05:01	C*16:01
7	A*03:01	am	B*07:02	B*51:01	C*02:02	C*07:02
8	A*03:01	am	B*57:01	B*44:02	C*07:04	C*12:03
9	A*03:01	am	B*07:02	B*14:01/02	C*08:02	C*07:02
12	A*01:22N	A*02:01	B*39:01/06	B*44:02	C*17:01	am
13	A*03:01	A*01:01	B*35:08	am	C*04:01	am
14	A*01:01/22N	A*03:01	B*27:06	B*08:01	C*03:03/04	C*07:01/07
16	A*03:01	A*23:01	B*81:01/02	B*35:05/20	C*04:01	am
17	A*03:01	A*01:22N	am	am	C*07:02	am
18	A*01:22N	A*03:01	B*44:02	B*51:01	C*15:02	C*01:02
19	A*03:01	A*01:01	B*14:02	C*07:01/06	C*08:02/04	am
20	A*29:01	am	B*07:02	B*44:02	C*05:01	C*07:02
21	A*02:01	A*03:01	B*07:02	B*15:01/32	C*03:04	C*07:02
22	A*01:22N	am	B*08:01	B*15:11/12	C*03:04	am
23	A*01:22N	am	B*44:02/05	am	C*08:02	C*15:02
24	A*03:01	A*01:01/22N	B*57:01	am	C*06:02	C*14:02
29	A*03:01	am	am	am	C*04:01/03	C*07:02
30	A*03:01	am	B*44:02	B*14:06	C*08:02	am
31	A*01:22N	A*03:01	B*50:01	B*15:34/35	C*03:03/04	C*06:02
33	A*26:01	A*03:01	B*48:17	am	C*17:01	am
35	A*01:22N	am	am	am	C*08:02	C*17:01
36	A*03:01	am	B*08:01	am	C*12:03	C*07:01
37	A*03:01	am	B*02:02/20	B*35:03	C*07:02	C*04:01
38	A*01:22N	am	B*44:02	B*57:01	C*06:02	am
39	A*03:01	A*24:02	B*35:02/87	am	C*07:01	am
41	A*03:01	A*24:02	B*51:01	B*35:03	C*16:01	C*12:03
48	A*03:01	A*01:22N	am	B*40:10	C*01:02	C*03:04
49	am	am	B*44:05/31	am	C*03:03	am
50	A*03:01	am	B*08:01	am	C*12:02	C*07:01

**52**	A*01:01/22N	am	B*08:01	am	C*07:04	am
**53**	A*29:01	am	B*35:10/20	am	C*04:01	C*06:02/30
**54**	A*03:01	A*32:01	B*15:39/197	B*40:02	C*02:02	C*07:02/04
**58**	A*03:01	A*11:01/50Q	B*07:02	B*18:01/02	C*07:01	C*07:02
**59**	A*03:01	am	B*57:01	B*37:01	C*06:02	am
**60**	A*24:02	am	B*14:02/06	B*35:02	C*08:02	C*16:01
**66**	A*03:01	am	B*44:01	am	C*02:02	C*15:02
**70**	A*03:01	A*01:01/22N	B*07:02	am	C*07:02	C*03:04
**71**	A*03:01	A*26:01	B*40:01	am	C*03:03/04	C*04:01
**73**	A*02:01/06	A*01:22N	B*07:02	B*40:01	C*07:02	C*03:04
**74**	A*26:01	am	B*55:01/02	am	C*03:04	C*12:03
**75**	A*29:01/02	am	B*51:42	am	am	am
**78**	A*03:01	am	B*07:02	B*35:01/31	C*02:02	C*07:01/02
**80**	A*23:01	am	am	am	am	am
**83**	A*01:22N	am	B*07:02	am	C*07:02	am
**84**	am	am	B*18:01	am	C*02:02	C*07:01
**86**	A*03:01	A*29:01	B*35:02	B*57:01	am	am
**91**	A*01:01	A*26:01	B*08:01	B*35:11/31	C*07:01	am
**92**	A*03:01	am	B*35:01/21	B*50:01	C*04:01/03	C*07:02/16
**95**	am	am	B*44:05	am	C*02:02	C*08:02
**96**	A*01:01	A*03:01	B*07:02	B*57:01	C*06:02	C*07:02
**99**	A*03:01	am	B*07:02	am	C*07:02	C*03:03/04
**100**	A*03:01	A*24:02/08	B*15:17	am	C*07:01	C*12:03
**101**	A*29:01/02	A*01:22N	B*38:01/02	am	C*16:01	am
**102**	A*26:01	A*03:01	B*35:01	am	C*04:01	C*06:02
**104**	A*29:01	am	B*07:02	am	C*07:02/51	C*16:01
**106**	am	am	B*73:01	am	C*15:05	C*15:02
**110**	A*01:01/22N	am	B*40:01	B*37:01	C*06:02	am
**118**	A*01:01/22N	A*03:01	B*08:01	B*44:02	C*05:01	C*07:01
**119**	am	am	B*40:01/02	B*13:02	C*08:02	C*06:02

Supplementary Table 2. HLA typing of patient samples. HLA-A, B, and C alleles were determined using Sequ2HLA. Some patient HLA types were confidently determined at the allele group, but prediction of the specific HLA protein was ambiguous between tumor and non-tumor samples. Such results are indicated by listing both predicted HLA proteins separated by /. Other alleles were entirely ambiguous (am) and are noted as such.
